# Life history traits of spotted lanternfly (Hemiptera: Fulgoridae) when feeding on grapevines and tree of heaven

**DOI:** 10.3389/finsc.2023.1091332

**Published:** 2023-02-22

**Authors:** Erica Laveaga, Kelli Hoover, Flor E. Acevedo

**Affiliations:** Department of Entomology, The Pennsylvania State University, University Park, PA, United States

**Keywords:** spotted lanternfly, grape, development, mortality, reproduction, fitness, tree of heaven, concord

## Abstract

The invasive planthopper, spotted lanternfly (SLF), *Lycorma delicatula* (White) (Hemiptera: Fulgoridae), feeds on a broad range of plants including species of economic importance such as grape. Although SLF feeds on wild and cultivated grape, the effect of grapevines on the insect’s life history traits is unknown. This study examined the effect of cultivated Concord grapevines (*Vitis labrusca*) and the insect’s preferred host tree of heaven (TOH), *Ailanthus altissima*, on SLF development, survival, reproduction, and body mass. Newly emerged nymphs were allowed to feed on either TOH, Concord grapevines or a mixed diet of Concord grapevines plus TOH through adulthood until death. Development, mortality, and oviposition of paired adults were tracked daily to calculate the SLF rate of development, survival, and reproduction among treatments. When feeding exclusively on Concord grapevines, SLF was able to develop and reproduce but had higher mortality, slower development, and produced fewer eggs. SLF fed on the mixed diet of grapevines plus TOH exhibited faster nymphal development, laid more eggs, and had higher body mass compared with those fed only on grape or TOH. SLF had greater survival when fed on either the mixed diet or on TOH alone. We conclude that Concord grapevines are a poor-quality host for SLF, but when combined with TOH, SLF fitness increases above that of feeding on TOH alone. This study supports the elimination of TOH as a part of SLF vineyard management practices.

## 1 Introduction

*Lycorma delicatula* (White) (Hemiptera: Fulgoridae), commonly known as the spotted lanternfly (SLF), is an invasive planthopper introduced into the United States. SLF is native to southeast Asia and was first detected in Berks County, Pennsylvania (PA) in 2014 (1). Despite efforts to control and contain its populations, SLF has spread to numerous states in the Northeast, Mid-Atlantic, and Midwest regions of the U.S. The insect is highly polyphagous and can reach high numbers making it difficult to control. In Asia, 73 plant species within 32 families have been reported as hosts for SLF nymphs and adults (2). Worldwide, SLF has been reported in association with over 100 plant taxa, many of which are crops, representing a threat to U.S. agriculture, especially grapevines (3).

SLF’s preferred host is *Ailanthus altisimma*, commonly known as tree of heaven (TOH), which is a deciduous invasive tree native to China and first introduced to the U.S. as an ornamental species in Philadelphia, PA in 1784 (4). Other common hosts include *Acer* spp. (maple), *Juglans nigra* (black walnut), *Salix* spp. (willow), and *Vitis* spp. (grapevines) (5, 6). Despite its broad host range, SLF seems to be particularly detrimental to TOH and grapevines. High numbers of SLF individuals have been observed in vineyards in early fall in the SLF quarantine zone of Pennsylvania (7). SLF harms plants directly by feeding on phloem sap and indirectly by excreting honeydew leading to the growth of sooty mold (5). SLF causes loss of plant vigor and inhibition of photosynthesis in its host plants (6, 8). Extensive SLF feeding suppresses photosynthesis, sap flow and carbohydrate storage in grapevine roots compromising vine health (9). Economic losses in vineyards are associated with reductions in yield, increased use of insecticides for SLF control, and vine decline (7, 9).

SLF is univoltine; adults lay eggs in the fall from September to the first hard freeze. The eggs overwinter and hatch in the spring; the resulting nymphs undergo four nymphal stages before reaching adulthood in July and August. After mating and undergoing reproductive maturation for several weeks, female SLF lay egg masses on a variety of surfaces, including tree trunks, plant stems, posts, rocks, vehicles, and outdoor equipment (2, 10). Eggs can easily be moved by humans to other geographical regions aiding dispersal to distant sites (10). It is unknown how many egg masses can be laid by one mated female in its lifetime and the length of their preoviposition period. However, it has been reported that each SLF female can lay at least two egg masses before the first frost, and each egg mass contains between 20-50 eggs (2, 10). The duration of the nymphal stages is likely to vary with local environmental conditions due to the strong influence of temperature on insect development (11, 12). The optimal growing temperatures for SLF are 15-30°C and the growing degree days (GDD) required for development into their second, third, fourth instars, and adults have been calculated as 166.6, 208.7, 410.5, and 620, respectively (11). The base threshold temperature is the minimum temperature needed for an insect to develop; the base temperature for SLF has been calculated to be 10.4°C for egg development (12) and about 13°C, 12.43°C, 8.48°C, and 6.29°C for first through fourth instars, respectively (11).

Besides temperature, host plant diet also affects SLF development and life cycle duration (13). For several years after introduction to the U.S, it was assumed that SLF could not survive and reproduce without TOH. However, recent studies showed that SLF can complete its life cycle without TOH and reproduce on other hosts, including grapevines (8, 13, 14). SLF nymphs successfully develop into adults when fed on single diets of TOH and black walnut (13). Similarly, mixed diets of TOH and either apple or black walnut support SLF development to adulthood and reduce time of development (13). In wild conditions SLF nymphs and adults are often found on TOH, multiflora rose (*Rosa multiflora*), and grape (*Vitis aestivalis*) suggesting that these may be preferred hosts for different life stages (15). However, under controlled conditions, grapevines (*Vitis rotundifolia* Var Carlos) seem to only support SLF development to the fourth instar. Despite the economic importance of the grape and wine industry in the U.S., assessed at several billion dollars, the effect of commercial grape cultivars on SLF life history traits has not been investigated.

The goal of this study was to determine the effects of host plant diet on SLF life history traits using TOH and grapevines (*Vitis labrusca*) as single hosts and in combination as a mixed diet. We measured development rate in days and GDD, mortality rate, and reproductive success as the number of eggs laid, hatch rate, and adult dry mass. We hypothesized that SLF fed on mixed diets of grapevines plus TOH would have a shorter development time, lower mortality rate, higher reproductive success, and greater dry mass than when fed on either host alone. The findings of this study contribute to our current knowledge of SLF biology and may help with the design of SLF management strategies in vineyards.

## 2 Methods

### 2.1 Research site

This study was carried out under field conditions in Alburtis, PA within the Pennsylvania SLF quarantine zone from May to November of 2021. The field site was located at coordinates 40° 26’ 43.368’’ N, 75° 37’ 34.752’’ W in an area of approximately 1,200 m^2^ of land surrounded by trees and shrubs. Most trees near the field site were *Juglans nigra* (black walnut) and *Carya illinoinensis* (pecan). Adjacent to the site was a pond, a corn field, and cattle. The ground was covered by grass, over which black weed barrier (FLARMOR Pro Garden, 20 x 40 m) was placed to prevent grass overgrowth.

### 2.2 Plant material

Seeds of *Ailanthus altissima* were collected in the fall and winter of 2017-2020 from wild trees in State College, PA. The seeds were sown in a germination tray (25.4 x 50.8 cm with drain holes, Tru Leaf Market, Salt Lake City, UT) with growth media mix [Sunshine Mix 4 (peat moss, perlite, starter nutrient charge, dolomitic limestone, and long-lasting wetting agent), Sungro Horticulture, Agawam, MA]. The first set of 1,000 seeds sown in February 2021 were placed in a tray without seed alteration. The next set of 1,000 seeds sown in April had the seed coat manually removed by gently peeling the outer skin. Seeds sown with the seed coat removed had a higher percentage germination than seeds with the seed coat intact: 17.6% and 5.3%, respectively. Seeds with the seed coat intact germinated after 4 weeks, while seeds without the seed coat germinated within 2 weeks. Seedlings of about 10 cm in height were transplanted into 11.43 cm pots (Greenhouse Megastore, Sacramento, CA). TOH plants of about 20 cm tall were transplanted again into 9.46-liter pots (Greenhouse Megastore, Sacramento, CA) at Berks County, PA in June 2021. The growth media in the 9.46-liter pots consisted of a mixture of Sunshine Mix 4 and topsoil (Scotts Premium, Home Depot, State College, PA) at a 2:1 ratio. Plants were fertilized with 37 g of Osmocote plus (N:15, P:9, K:12), plus micronutrients six weeks after germination. Each plant was further supplemented once with a 500 ml solution of 10% chelated iron and 8% nitrogen (Sequestrene Iron 330 Fe, ProSolutions LLC, Maryville TN) 10 weeks after germination. The solution was prepared by diluting 4 g of the fertilizer in 4L of water. Bare root canes of Concord grapevines (*Vitis labrusca*) of ~2.5 cm stem diameter were purchased from Amberg Grapevines, LLC (Clifton Springs, NY) and planted in April of 2021. The vines were planted in 9.46-liter pots containing growing media (Sunshine Mix 4) and topsoil (Scotts Premium) in a 2:1 ratio. The vines were fertilized as described for TOH above. Grapevines of ~ 30 cm tall were used for the experiments in late May; subsequently, the vines were pruned regularly to a height of ~35 cm and fruit clusters were removed as they developed. TOH and grapevine plants were grown from February to May under greenhouse conditions (14:10 h of light: dark) at the Pennsylvania State University, University Park, PA. In early June, the plants were transported to Alburtis, PA for the experiments.

### 2.3 Insects

SLF egg masses were wild collected from Blue Marsh, PA (40° 23’ 60’’ N, 76° 4’ 11.99’’ W) in March 2021. The egg masses were either scraped off trees by cutting underneath the bark with a sharp knife or the masses were collected from smaller branches that were cut into pieces. The egg masses were then stored in plastic storage bins (79 x 51 x 38 cm) in a cooling chamber kept at 4°C for 60 days. After removal from the cooling chambers, the egg masses were placed in mesh cages [(90 x 60 x 60 cm), Jinhua Quiangsheng Outdoor Products, Zhejiang China] with TOH plants in ambient conditions at the research site for 3 weeks until nymphs emerged. Freshly emerged SLF nymphs were collected daily and immediately placed into their designated treatment cages.

### 2.4 Survivorship and development of spotted lanternfly in grape and tree of heaven

Newly emerged SLF nymphs were transferred to mesh cages (90 x 60 x 60 cm) containing one of the following plant treatments: TOH, Concord grape, or Concord plus TOH. Each cage was infested with five first-instar SLF nymphs that hatched the same day. The survival and development of SLF individuals from each cage was recorded every day until death. Throughout the season, plants were monitored for disease and replaced as needed to sustain the SLF individuals. Once the nymphs emerged as adults, individuals coming from the same plant treatment were paired into male and female couples and isolated in a cage containing the same combination of plants in which the nymphs developed. Grapevine and TOH plants used for adult feeding were ~45 cm tall and 5-months old from the time they were transplanted. Dead SLF males were replaced with new ones from the same plant treatment. Dead female adults were not replaced after oviposition. Adult survival was monitored until adults died naturally when temperatures reached 0 °C.

### 2.5 SLF oviposition

We recorded the number of SLF couples that laid egg masses, the number of egg masses laid by each SLF couple until first frost, the number of eggs within each egg mass, and the number of nymphs that hatched from those eggs. Within each cage of adult pairs, a polywood (7 x 60 cm) substrate was added for oviposition. SLF females laid their egg masses on either the Polywood, the side of the mesh cages, or on the plant itself. Egg masses laid on plants were collected by cutting the plant piece where they were laid, while egg masses laid on the cages were carefully scraped out and placed into 50 ml plastic tubes covered with mesh lids to allow air flow. Eggs laid on the polywood were left on that substrate and placed in plastic bins (79 x 51 x 38 cm). The egg masses were stored in a cooling chamber for 6 months at 4 °C.

### 2.6 SLF egg mass hatch

The collected egg masses were removed from the cooling chamber and acclimated to the ambient temperature in mesh cages (90 x 60 x 60 cm) in a greenhouse in April 2022. The number of eggs per egg mass was counted under a stereoscope (SZ30, Olympus, Tokyo, Japan) after gently brushing over the protective wax layer with a wet paper towel to reveal the eggs underneath. The number of hatched SLF nymphs were counted and divided by the number of eggs laid to calculate the percent of egg hatch. Hatch rate was recorded to document a successfully completed life cycle of the adult pairs.

### 2.7 SLF adult weight gain

Weight gain was determined for each SLF individual from the survivorship experiment that successfully developed into an adult. The adults were collected as they died, placed individually in properly labelled 5 ml tubes (Thermo Scientific) and stored at 4°C. Subsequently, each SLF adult was placed in a paper bag (7.6 x 5.1 x 15.2 cm) and dried in an oven at 60°C until their weight remained constant. The weight of each specimen was determined using an analytical scale accurate to 0.1 mg (Ohaus Adventurer™ Analytical Balance model AX124/E). The weight of each adult was standardized by the number of GDD it accumulated before dying; the standardized dry weight values were used for the statistical analyses.


$$Standardized\;Dry\;Mass=\frac{Adult\;SLF\;Mass\;\left(mg\right)}{Adult^{\prime }s\;Total\;GDD}$$

### 2.8 Weather data

Temperature (°C), humidity (%), and rainfall (mm and mm/h) were recorded daily at the research site using a Davis 6152 wireless Vantage Pro2 Weather Station (Scientific Sales Inc. Lawrenceville, NJ. USA). Measurements with the weather station began June 30, 2021. Temperature data prior to June 30 was collected using Weather Underground weather history (TWC Product and Technology LLC 2014, 2022).

### 2.9 Experimental design and data analysis

To determine differences in development and survival of SLF nymphs on different plant diets, each experimental unit was comprised of five nymphs enclosed in a mesh cage with its respective plant treatments. For adults, the experimental unit comprised one couple (male and female) enclosed in a mesh cage with the same plant treatment in which they developed as nymphs. The experimental units (cages) were set up in a completely randomized design at the research site

#### 2.9.1 Development

SLF development was analyzed by calculating the number of days and the number of GDD required for each nymph to molt into the next developmental stage (instar or adult) using the following formula described by Herms (16).


$$GDD=\left(\frac{Max\;temperature+Base\;temperature}{2}\right)-Base\;temperature$$

GDD calculations that resulted in a negative value were replaced with 0. Calculating the GDD using the average of the maximum temperature and base temperature (Modified Average Method) has been reported to be more accurate than the average of the maximum and minimum temperatures (Average Method) because it accounts for periods of time when the temperature is above the base threshold even if the average temperature is below it (16). Development still occurs when the average temperature is below the base threshold if the maximum temperature surpasses the base temperature (16). The base temperatures used for calculating GDD for first through fourth instar nymphs were 13.00°C, 12.43°C, 8.48°C, and 6.29°C, respectively (11). GDD were summed for each individual per instar to obtain the accumulated GDD. We averaged the number of days and the number of GDD it took the nymphs within each experimental unit to develop into their next stage; this value was used as an independent replication for statistical analyses. Differences among treatment means for the GDD per instar and the number of days spent in each instar were analyzed with one-way Analysis of Variance (ANOVA). Significant differences between treatment means were elucidated with the Tukey test at alpha = 0.05. GDD data from first, fourth, and total instars were transformed using inverse squared. For the second instar we used the inverse transformation, and for the third instar we used an inverse square-root transformation to meet the assumptions of normality and equal variances before pursuing the ANOVA. Data for development time in days were transformed using the inverse for the first and second instar, log base 10 transformation for the third, and inverse square root transformation for the fourth instar to meet the assumptions of normality and equal variances.

#### 2.9.2 Survival

We calculated the percentage of nymphs that survived per instar and the percentage of adults that survived from emergence to first frost for each experimental unit. Each data point from an experimental unit was used as an independent replicate. Differences in survival rates per treatment and SLF biological stage were analyzed using a generalized linear model (GLM) that best fitted the error distributions of proportion data. We fitted a binomial model with a logic link function and tested the significance of the model terms using an analysis of deviance. Overdispersion was tested using the deviance and Pearson Goodness of Fit tests (17); in the presence of overdispersion, a quasibinomial model was fitted (17). Multiple comparisons between treatment pairs were assessed using the glht-tukey method implemented in the multcomp R package (18). In addition, we constructed Kaplan-Meier survival curves for the nymphal stage (first to fourth instar) to better visualize the survival probability of SLF feeding on different plants. When all the nymphs within a cage died, that experimental unit was registered as dead or 1 in the data base, while experimental units with nymphs alive were evaluated as “censored” or zero in the data base. To calculate the time to death, we averaged the days alive of each nymph per instar within each experimental unit and used that value for the K-Meier model. Statistical differences among treatments were determined using the log-rank test (19).

#### 2.9.3 Life table analysis

The number of days SLF spent in each instar was used to construct a life table. Life table analysis displays the proportion of experimental units alive in each treatment at the beginning of each life stage or instar. The probability of surviving the period was calculated by the average proportion of experimental units alive by the end of each life stage divided by the number of experimental units alive at the start of the life stage. Percent probability of death was calculated using the average percent mortality for experimental units within each life stage. Cumulative number of days of survival beyond each life stage (*Age* * *Tx*) was the average cumulative survival days of each experimental unit.

#### 2.9.4 Oviposition

To assess the effect of each plant treatment on SLF oviposition, we calculated the percentage of couples that laid egg masses out of the total number of initial pairs, the average number of eggs laid per egg mass, and the percentage of nymphs that hatched from those eggs. When a single female laid more than one egg mass, data were averaged for that female. Data for couples that came from the same experimental units in their nymphal stage were averaged and the resulting number used as an independent datum for the statistical analyses. The number of experimental units for adult SLF couples was 51 for TOH treatment, 8 for Concord, and 30 for Concord plus TOH treatment. From these, the total number of independent replications per treatment was 45 for the TOH treatment, 4 for Concord, and 26 for Concord plus TOH. Differences in the average number of eggs laid per egg mass and the total number of eggs per treatment were assessed with one-way ANOVA followed by the Tukey test. Differences in the percentage of nymphs that hatched between treatments were analyzed using Chi-square. The number of GDD and days from female emergence to the first egg mass laid (preoviposition period) were calculated as explained above for SLF development. Differences among treatment means were analyzed with one-way ANOVA followed by a Tukey test at alpha = 0.05.

#### 2.9.5 Weight gain

The dry weight of SLF adults was standardized by dividing the individual’s weight by the total GDD accumulated by each adult using the base temperature of 10.4 °C (12). The standardized data were then analyzed using a one-way ANOVA followed by a Tukey test at alpha = 0.05.

## 3 Results

### 3.1 Spotted lanternfly development

SLF nymphs feeding on Concord grapevines developed slower than nymphs feeding on Concord plus TOH or TOH alone (**Table 1**). The average number of GDD required for nymphal development across treatments increased gradually from first to fourth instar [mean ± SEM:123.42 ± 1.83 (n=120), 136.1 ± 2.3 (n=116), 214.4 ± 5.3 (n=105), 296.7 ± 7.3 (n=81), respectively] for all treatments. There were no significant differences in the number of GDD and development time in days between treatments for the first instar (**Table 1**, rows 2-4). Nymphs feeding on the single-host Concord diet began to display significantly slower development (required more days and GDD to molt) by the second instar compared to the single-host TOH or the mixed-host diet of Concord plus TOH (**Table 1**, rows 5-7). SLF feeding solely on Concord vines required on average between 3.7 to 6.1 more days to develop into the third instar, and between 3.5 to 13.1 more days to develop into their fourth instar than those fed on mixed-host diets or TOH alone. Second instars fed on Concord alone required two more days to develop than those fed on mixed-host diets (**Table 1**). The total number of GDDs required to develop from first instar to adult eclosion were between 146 to 206 greater when fed on Concord compared with other diets, but the development time in days did not differ statistically among treatments (**Table 1**). Overall, SLF individuals fed on Concord grapevines required the greatest number of GDDs to develop through the nymphal stages (894.2 ± 34.3), whereas individuals feeding on the mixed-host diet of grape plus TOH required the fewest GDDs (688.2 ± 14.9).

**Table 1:** Average growing degree days and number of days required for each spotted lanternfly (SLF) instar to develop when fed on Concord grape, tree of heaven (TOH), or the combination of Concord and TOH.

SLF Instar	Plant Treatment	N	Average GDD ± SEM	Average development time (days ± SEM)	df	GDD		Development time (days)	
						F-value	P-value	F-value	P-value
First	Concord + TOH	26	121 ± 3.4a	16.7 ± 0.4a	2, 117	2.48	0.089	1.22	0.299
	TOH	47	120.9 ± 3.3a	16.5 ± 0.4a					
	Concord	47	127.3 ± 2.6a	17.0 ± 0.3a					
Second	Concord + TOH	25	126 ± 3.2a	13.9 ± 0.4a	2, 113	6.73	0.002	5.01	0.008
	TOH	46	132.5 ± 3.9a	14.9 ± 0.4ab					
	Concord	45	145.3 ± 3.6b	15.9 ± 0.4b					
Third	Concord + TOH	23	178.2 ± 5.4a	17.6 ± 0.6a	2, 102	18.29	<0.0001	15.83	<0.0001
	TOH	45	205.4 ± 7b	20.0 ± 0.7a					
	Concord	37	248 ± 9.4c	23.7 ± 0.7b					
Fourth	Concord + TOH	23	265.3 ± 10.1a	22.5 ± 0.9a	2, 78	12.33	<0.0001	24.9	<0.0001
	TOH	45	290.6 ± 7.5b	26.0 ± 2.4b					
	Concord	13	373.6 ± 22.4c	35.6 ± 0.8c					
Total	Concord + TOH	23	688.2 ± 14.9a	70.5 ± 1.6a	2, 78	19.48	<0.0001	0.09	0.912
	TOH	45	748.9 ± 14.5b	77.4 ± 1.6a					
	Concord	13	894.2 ± 34.3c	90.8 ± 3.8a					

Different letters indicate significant differences among treatment means obtained with the Tukey test at alpha=0.05 following ANOVA. N= number of experimental units, GDD= number of growing degree days, SEM= Standard error of the mean, df= degrees of freedom (treatment, error), F-values and P-values were obtained with one way ANOVA.

### 3.2 Survival

SLF survival varied at different stages of development and by host-plant diet. The average survival of nymphs across treatments was 64.88% for first instars, 90.87% for second instars, 89.48% for third instars, and 82.2% for fourth instars (**Table 2**). SLF survival was also affected by host plant diet; third and fourth instars had significantly lower survival when fed on Concord grapevines alone compared to those fed on either TOH or the mixed-host diet of grape plus TOH (**Table 2**). The average survival rate of SLF nymphs from first instar to adult emergence was lowest on Concord (6.3%) compared with TOH (37.7%) and the mixed host diet [(50.6%), (**Table 2**, rows 14-16). The average survival of adults to the first frost in November 2021 was 58.36% across treatments. Adults fed on Concord had the lowest survival rate compared with those fed on either TOH or the mixed host diet treatment (**Table 2**, rows 17-19). Adult SLF individuals feeding on Concord alone also had the shortest life span before the first frost of the season (17 ± 5.1, n=6) compared with those feeding on TOH alone (47.4 ± 2.5 days, n=40) and the mixed host diet (42.5 ± 6 days, n=16), ANOVA F_2,59 =_ 7.26, P<0.05)]. Overall, the lowest survival rates across treatments were for adults and first instar nymphs. The highest survival rates of SLF nymphs and adults were for individuals fed on the mixed host diet and the TOH treatments (Tale 2, column 6).

**Table 2:** Survival of SLF nymphs and adults when fed on Concord grape, TOH, or the combination of grape and TOH. The “Initial No. of nymphs” describes the total number of individual nymphs in each treatment at the beginning of the experiment.

Instar	Plant Treatment	Initial No. of nymphs	Initial No. of Experimental Units	Experimental Units Alive (%)	SLF Survival (%)	df	F/Z Values	P- Value
First	Concord + TOH	132	26	100	68.59a	2, 124	0.853	0.428
	TOH	255	51	100	60.4a			
	Concord	251	50	100	65.67a			
Second	Concord + TOH	90	26	100	95.52a	2, 117	2.3159	0.103
	TOH	154	47	92.157	89.79a			
	Concord	165	47	94	87.31a			
Third	Concord + TOH	84	25	96.2	95.2a	2, 114	13.174	7.12e-06
	TOH	130	46	90.2	94.4a			
	Concord	135	46	92	78.84b			
Fourth	Concord + TOH	78	23	88.5	91.3a	2, 101	-7.1366	9.5e-13
	TOH	117	44	86.3	93.2a			
	Concord	87	37	74	62.26b			
Average Nymph Survival Rate	Concord + TOH	132	26	88.46	50.6a	2, 124	26.121	3.41e-10
	TOH	255	51	86.27	37.7a			
	Concord	251	50	24	6.3b			
Adults before first frost	Concord + TOH	66	23	88.5	72.7a	2, 76	-3.5488	3.87e-04
	TOH	104	44	86.3	58.7a			
	Concord	16	12	24	43.7b			

The “Experimental Units Alive (%)” describes the percentage of experimental units remaining in each instar with at least one SLF individual alive. “SLF survival (%)” represents the percent of individuals that survive per life stage out of those that molted into that stage. Mortality rates of each nymphal instar and adults were analyzed using a generalized linear model (GLM). Different letters indicate significant differences among treatments using the *post hoc* glht-Tukey test implemented in the multcomp R package (17). df =degrees of freedom (treatment, error), F/Z: F-values obtained from fitting quasi-binomial models and Z values were obtained from fitting binomial models.

**Figure 1:**
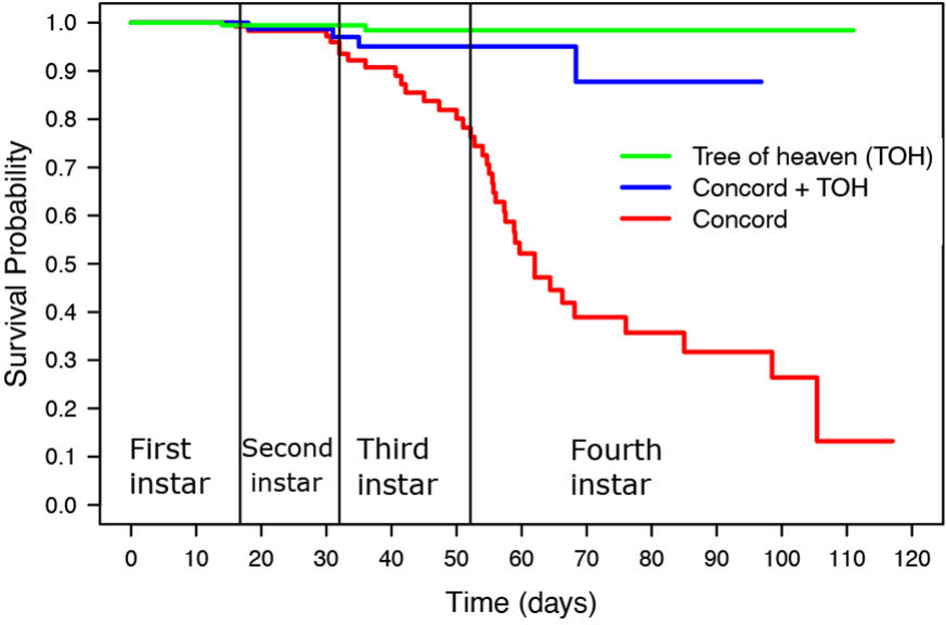
Kaplan Meier survival curves of SLF nymphal instars fed on tree of heaven (TOH), Concord, and Concord plus TOH.

The Kaplan-Meier Survival Curve (**Figure 1**; **Table 3**) shows the SLF survival probability throughout the four nymphal instars. SLF fed on TOH and the mixed diet of grape plus TOH had a cumulative survival rate above 80% throughout all four nymphal stages while SLF fed on Concord reached 50% survival probability before day 60, which occurred in the fourth instar (**Figure 1**). The log rank test showed significant differences in survival probability between SLF fed on the mixed diet and Concord (χ^2 =^ 17.1, P<0.05), and between nymphs fed on Concord and those fed on TOH (χ^2 =^ 52.4, P<0.0001). There were no significant differences in survival probability between the mixed diet treatment and TOH alone (χ^2 =^ 3.1, P >0.05).

**Table 3:** Life table comparison of SLF in each instar fed on either Concord, TOH, or Concord plus TOH plants.

Treatment	Instar	Proportion of Individual Surviving (Lx)	Probability of Surviving the Instar (Npx)	lx*px	Percent Probability of Death 100 qx	Cumulative Number of Days Lived beyond Age*Tx
Concord + TOH	First	1.00	0.686	0.69	31.41	96.65
	Second	0.69	0.955	0.66	4.48	82.71
	Third	0.66	0.952	0.62	4.80	65.07
	Fourth	0.62	0.913	0.57	8.70	42.56
TOH	First	1.00	0.604	0.60	39.60	108.31
	Second	0.60	0.898	0.54	10.21	93.54
	Third	0.54	0.944	0.51	5.60	73.41
	Fourth	0.51	0.932	0.48	6.80	47.40
Concord	First	1.00	0.657	0.66	34.33	92.36
	Second	0.66	0.873	0.57	12.69	76.38
	Third	0.57	0.788	0.45	21.16	52.65
	Fourth	0.45	0.623	0.28	37.74	17.00

The total proportion of experimental units alive at the beginning of each instar (Lx), Npx describes the survival probability in each instar, lx*px depicts the proportion of experimental units alive to the total initial experimental units, the “Percent Probability of Death 100 qx” is the percent mortality per instar, and the cumulative number of days SLF is alive after each instar is denoted by Age*Tx.

A life table summarizes the cumulative probability of survival at the beginning of each instar (Lx) and the probability of surviving the instar [(Npx), (**Table 3**)]. SLF nymphs feeding on the mixed diet consistently had the highest probability of survival in their first to third instar, whereas fourth instar nymphs had a higher probability of survival when fed on TOH and the mixed diet treatment (**Table 3**, column 4). Nymphs fed on TOH alone also survived the greatest number of days after each consecutive instar (**Table 3**, column 7).

### 3.3 Spotted lanternfly reproduction

The pre-oviposition period (time from adult emergence to first egg mass laid) in SLF ranged from 30-50 days, which corresponded to 250-500 GDDs using a base temperature of 10.4°C (12) (**Table 4**, column 3). There were no significant differences in the number of days or GDD during the pre-oviposition period among treatments (F_3,52_ = 1.15, P = 0.338). The number of SLF females that laid at least one egg mass was greatest in the single diet of TOH. However, paired SLF females fed on Concord plus TOH laid the greatest number of egg masses [(column 4), (χ^2 =^ 21.221, df = 12, P = 0.04724). Similarly, SLF females fed on Concord plus TOH laid significantly more eggs than those fed on TOH alone (F_3,53_ = 5.16, P = 0.003; **Table 4**). Females fed on the mixed diet laid on average 2.58 egg masses and 94.89 eggs per female, whereas those fed on TOH laid on average 1.72 egg masses and 48 eggs per female. SLF females fed on Concord only laid one egg mass containing 45 eggs. The average number of eggs per egg mass ranged from 20 to 45 (**Table 4**). The number of first instar nymphs that hatched from these egg masses was <10.5% for all treatments with no significant differences in percent hatch among treatments (χ^2 =^ 29.87, df = 45, P = 0.9597, **Table 4**).

**Table 4:** Reproduction parameters of SLF individuals grown on Concord grape, TOH and Concord plus TOH.

Treatment	SLF Couples that Oviposited (%)	Pre-Oviposition		Total Egg Masses	Avg. Number of Eggs per Egg Mass ± SEM	Avg. Percent of egg Hatch
		Avg. GDD ± SEM	Avg. Days ± SEM			
Concord +TOH	73 (n=26)	384.5 ± 14.2	44.2 ± 1.51	49	34.9 ± 2.27	10.2
TOH	45 (n=49)	370.3 ± 16.5	43.5 ± 1.53	38	25.8 ± 2.12	5.7
Concord	9 (n=11)	168.2 ± 0	34 ± 0	1	45.0 ± 0	0

Average number of growing degree days (Avg. GDD) and average number of days (Avg. Days) from female emergence to oviposition; SEM= standard error of the mean.

### 3.4 Adult weight gain

**Figure 2:**
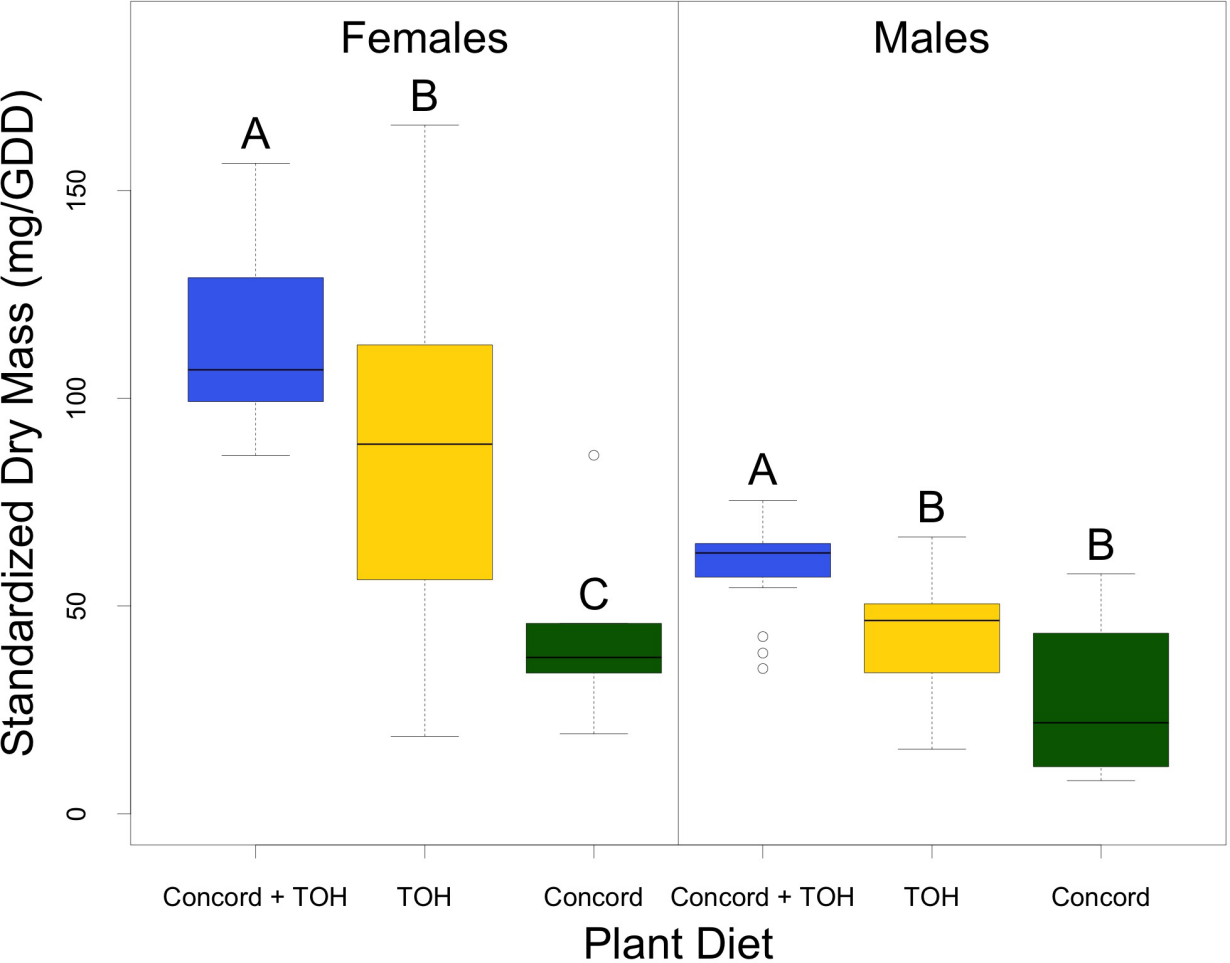
Weight gained by SLF adults fed on different diet treatments. Dry mass was standardized by dividing the raw dry mass by the growing degree days. Horizontal bars represent the medians, the box represents the interquartile range, the whiskers represent the range of the data scores, and dots outside of the plots are outliers. Differences among treatment means were analyzed with one way ANOVA. Differences between treatment pairs were analyzed with the Tukey test (alpha = 0.05) following ANOVA. Different letters indicate significant differences among treatment means. Data did not require transformations to meet the assumptions of normality and equal variances before doing the ANOVA.

Adult dry mass was influenced by host diet and gender (**Figure 2**). Females on average weighed 43 mg more than male adults. Females fed on Concord plus TOH had the highest dry mass (x̄ = 114.2 ± 4.9 mg, n= 17), followed by those fed on TOH alone (x̄ =85.3 ± 6.7 mg, n=35). Females fed on the single Concord diet had the lowest dry mass with an average of 44.6 ± 11.3 mg (F_2,54_ = 8.9, P< 0.001; n=5). Weight gained by male adults showed a similar trend to those of females; males fed on Concord plus TOH had significantly greater dry mass (x̄ =59.4 ± 2.6 mg; n=18) than males fed on TOH (x̄ =43.2 ± 2.3 mg; n=34) or Concord alone [(x̄ =27.4 ± 11 mg; n=4), (F_2,53_ = 13.71, P< 0.001)].

### 3.5 Weather data

The maximum temperature recorded at the Alburtis PA field site was 35.5°C while the minimum temperature recorded was -4.4°C, on August 13 and November 2, respectively. The maximum temperatures above 33°C occurred in the months of July and August 2021. The minimum temperatures below 0°C occurred in the first week of November 2021 (**Supplementary Figure 1**). Daily rainfall recorded at the field site measured 5 peak rainfall days with over 20 mm of rain. Days with more than 20 mm of rainfall were August 18 and 22-23, September 1 and 23, and November 3, 2021 (**Supplementary Figure 2**). Hurricane Ida was a category 4 Atlantic hurricane that affected Pennsylvania August 30-September 5 as a tropical storm. The storm hit the field site on September 1 with total rainfall of 56.9 mm for the day. Maximum percent humidity consistently ranged from 90-100% while the minimum percent humidity varied between 34 and 96%. Peaks in minimum percent humidity occurred simultaneously with rainfall (**Supplementary Figure 3**).

## 4 Discussion

The results of this study show that host plant diet has a strong influence on SLF fitness and biology. SLF individuals were able to develop and reproduce when feeding exclusively on Concord grapevines; however, there was high percentage mortality of nymphs from the first instar to adult emergence (93.66%), development was slower for these nymphs, and adults laid fewer eggs than those feeding on a mixed diet or on TOH alone. SLF individuals feeding on a mixed diet of grape plus TOH had faster development to adulthood and laid more egg masses than those fed on single diets of either grape or TOH. At the nymph stage, survival was highest when feeding on TOH and the mixed diet.

Development rates also varied among diet treatments. SLF developed faster when fed on mixed diets of grape and TOH; there was no influence of diet on the development of first instars, but as the nymphs reached their second, third and fourth instar, there were significant differences in development between SLF feeding on different host plants. Nymphs fed on a mixed diet of grape and TOH developed faster than those fed on Concord alone. No significant differences in development were observed for first instar nymphs, possibly due to low nutritional requirements of that stage to enhance survival. Second instar nymphs developed the slowest when fed on grape as a single diet. In general, nymphs required the lowest GDD when fed on mixed diets and the highest when fed on Concord grape alone (**Table 1**). These differences in rates of development may relate to the nutritional quality of a mixed diet versus a single host diet (20). Studies have shown that TOH is a high-quality host plant for SLF (8, 14), which may be due in part to their shared native range and history of host plant preference or co-evolution (5). The GDD required for second-fourth instar nymphs to develop were lower than those reported in a previous study (11) regardless of the calculation method used, i.e., the Average Method (not shown), and the Modified Average Method (16). Thus, the lower GDD ranges found in this study were likely due to different experimental conditions, i.e., field vs laboratory, microclimate inside experimental cages, differences in humidity, or stress from other abiotic factors. The microclimate within the cages could have been slightly different from the temperature recorded by the weather station due to the mesh enclosure and placement of cages on a black weed barrier, which may have increased the microclimate temperature in the cages, and the mesh obstructs some of the airflow, raising temperatures. This and previous studies agree that SLF can develop without access to TOH, but their development time is slower, their mortality is higher, and their oviposition is reduced (6, 13, 14).

Host plant diet also affected SLF survival. The average survival rate and the survival probability of SLF nymphs was lowest when feeding exclusively on Concord grape, and highest when feeding on the mixed diet and on TOH. These results suggest that Concord grape alone is a poor diet for SLF compared with the other treatments. Our results agree with a previous study in which mixed diets of TOH plus either apple, black walnut, grapevine (*Vitis rotundifolia*, var. Carlos), or peach improved SLF survival compared with single host diets (13). There was a significant decrease in the survival probability for SLF fed exclusively on Concord grapevines by the third and fourth instars, while survival on TOH and mixed-host diets remained above 80% through the four nymphal stages (**Figure 1**). Survival probability was similar for SLF feeding on the mixed diet and on TOH alone (**Figure 1**). Various studies have demonstrated that mixed diets improve growth rates in polyphagous herbivores compared with less diverse diets (21) and SLF is a highly polyphagous insect, with a reported host range of at least 100 different plant taxa (3). Two hypotheses have been proposed to explain this phenomenon; (i) the nutrient balance hypothesis proposed by Pulliam (22) argues that a mixed diet allows herbivores to switch between diets with contrasting nutrient content; and (ii) the dilution of toxin hypothesis proposed by Freeland and Janzen (23), which argues that mixed diets allow for dilution of plant secondary metabolites by feeding on plant material with different allelochemical content (22, 23). Studies with various herbivore species strongly support the nutrient balance hypothesis (21, 24), whereas the effect of toxic plant allelochemicals seems to depend on the food nutrient composition (25, 26). SLF feeds on plant phloem for which nutrient compositions are known to vary among plant species and with abiotic factors, developmental stage, and time of the season (27). Further, SLF dispersal capabilities may allow the insect to regulate its nutrient intake by feeding on multiple hosts.

SLF survival also varied for different developmental stages. The lowest average survival rates across treatments were found in adults and first instar nymphs compared with second through fourth instar nymphs (**Table 2**). This is likely due to disparate nutritional requirements of different life stages and possibly variation in tolerance to secondary compounds found in their diet. SLF is known to vary in its host preference at different stages of development (2, 5). Although highly polyphagous, adults are known to narrow their host plant preferences compared to nymphs (15). Early instar nymphs have been observed to feed on young plant growth and on herbaceous plants, whereas adults seem to prefer woody host plants and tissues (3). When feeding on grapevines, early instar nymphs feed exclusively on shoots and the veins on the undersides of leaves. Third and fourth instars can feed on shoots and cordons, whereas adults feed on shoots, cordons, large branches, and tree trunks (7). Variation of feeding sites within a single plant species may be associated with morphological variations in SLF mouthparts at different stages of development, and with differences in plant sap flow rate through the growing season (7, 28). Besides, the effects of host plant diet and insect developmental stage, we did not find an effect of local environmental conditions on SLF mortality, except for the first frost that killed the adults on November 2-4 of 2021. Surprisingly, Hurricane Ida on September 1 had no effect on SLF mortality. The cages had fallen over from the strong winds, but there were no spikes in mortality on the days following.

The mixed diet also improved SLF reproduction compared with single host diets of either grape or TOH. Fertilized females fed on the mixed diet laid the greatest number of egg masses and total eggs followed by those fed on TOH. Our results show that access to a mixed host diet containing TOH doubles the number of eggs oviposited by females when compared to a single diet of TOH. From the SLF females that fed on just Concord grapevines, one of them oviposited a single egg mass, but none of these eggs hatched. Poor quality diet is linked to poor reproductive rate and low-quality eggs, which can also force early reproduction to ensure a next generation (29). The average percent of eggs that hatched was very low for all treatments, which may have been due to our experimental conditions. Low percent egg hatch could have been affected by premature placement of the egg masses into cooling chambers, the storage period and temperature, or the acclimation to greenhouse conditions. It has been reported that prolonged egg storage beyond one month at 5 °C decreases SLF egg hatch rate (30). In a field study conducted in Berks (Pennsylvania) in 2017, egg hatch ranged from 51.5 to 84.2%, but egg hatch seems to be highly dependent on winter temperatures (2). The time from female emergence to oviposition ranged from 4-6 weeks, which is similar to previous field observations (2), indicating that the insect has a relatively short time to lay eggs before the first frost in the northeast U.S. Although male and female SLF couples were put together in cages soon after emerging, we have no record on when mating occurred. The insects showed a visible increase in the size of their abdomens (not measured) before they started laying eggs. This suggests that egg production and maturation seem to require a large accumulation of body reserves through food consumption. The pre-oviposition time did not differ among females reared on different host diets; however, more research should be conducted to explore the effects of diet on duration of the SLF preoviposition period and oviposition rates.

Diet type had a strong effect on the body weight of SLF adults. Females reared on the mixed diet gained more weight than those fed on single host diets, and males gained more body weight when fed on the mixed diet compared with those fed on TOH or Concord alone (**Figure 2**). Body weight is an indicator of insect health (31) and is associated with the nutritional quality of their host plants (32, 33). Also, the high variance within treatments can be explained by the presence or absence of eggs within the female’s abdomen. Since the couples were actively laying egg masses at the time of death and sample collection, there may have been females that were unable to lay all their eggs or to mate. The ability to successfully mate can contribute significantly to the dry mass of both males and females due to the transfer of a large spermatophore from the male (5).

In summary, the results of this study show that SLF development, reproduction, and body mass benefit from a mixed diet with TOH compared to feeding solely on grapevines or TOH. SLF survival was highest when fed on either the mixed diet or on TOH. When feeding exclusively on Concord grapevines, SLF was able to develop and reproduce but its fitness was greatly reduced. Our results suggest that SLF management in vineyards could benefit from limiting access to TOH to reduce insect fitness, but more research is needed to compare variations of mixed diets on the insect’s life cycle.

## Data availability statement

The raw data supporting the conclusions of this article will be made available by the authors, without undue reservation.

## Author contributions

FA designed the study. EL conducted the experiments. EL and FA analysed the data and wrote the manuscript. KH contributed to the identification of the research site, logistics in experimental set up and provided valuable input to the manuscript. All authors read, contributed to revisions, and approved the manuscript.
