# Supplementary material for: Life history traits of spotted lanternfly (Hemiptera: Fulgoridae) when feeding on grapevines and tree of heaven
Source: Front Insect Sci. 2023 Feb 22;3:1091332. doi: 10.3389/finsc.2023.1091332 (PMC10926547; doi:10.3389/finsc.2023.1091332)
Supplement: Supplementary file 1 [file DataSheet_1.docx]

Supplementary Material

# Supplementary Figures

Supplementary Figure **1**: Maximum and minimum temperatures recorded at the field research site in Alburtis PA (coordinates 40.44, -75.63) from May to October 2021.


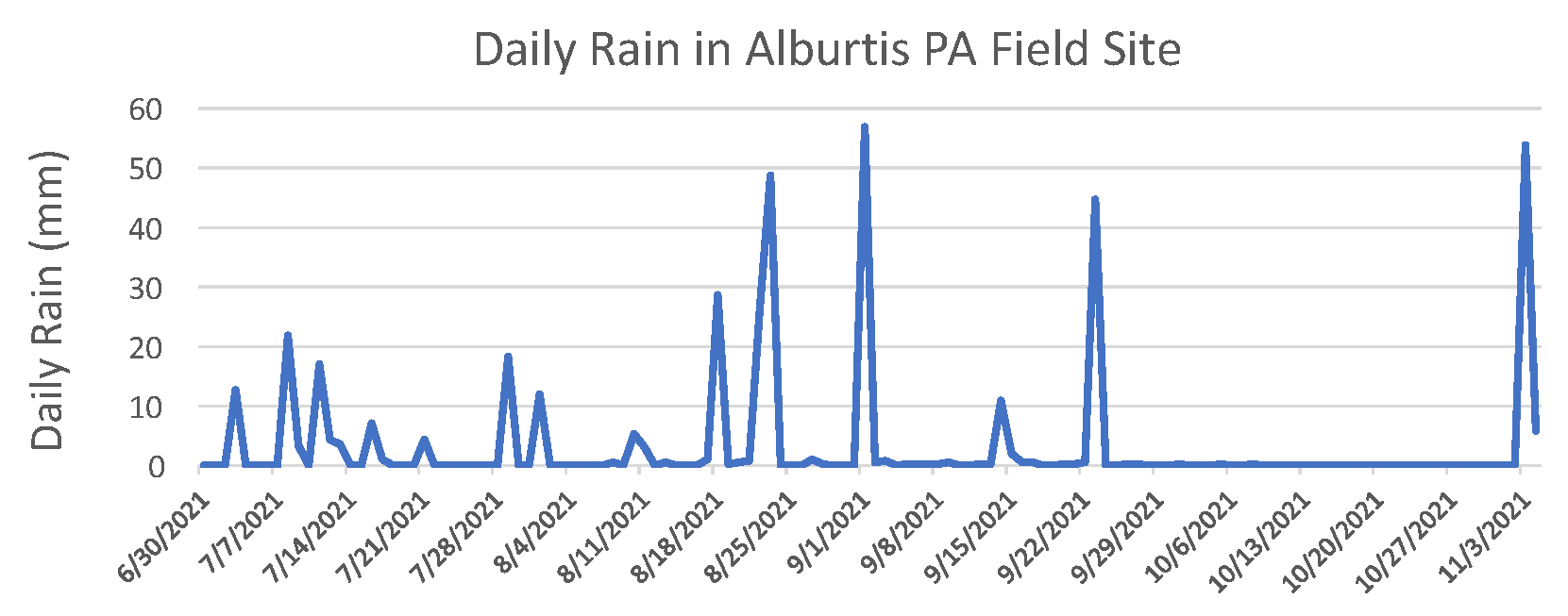


Supplementary Figure **2**: Daily rainfall recorded at the field research site in Alburtis PA (coordinates 40.44, -75.63) from May to October 2021.

Supplementary Figure **3**: Maximum and minimum percent humidity recorded at the field research site in Alburtis PA (coordinates 40.44, -75.63) from May to October 2021.
